# How Small Molecules Affect the Thermo-Oxidative Aging Mechanism of Polypropylene: A Reactive Molecular Dynamics Study

**DOI:** 10.3390/polym13081243

**Published:** 2021-04-12

**Authors:** Fan Zhang, Yufei Cao, Xuan Liu, Huan Xu, Diannan Lu, Rui Yang

**Affiliations:** Department of Chemical Engineering, Tsinghua University, Beijing 100084, China; zhangf19@mails.tsinghua.edu.cn (F.Z.); caoyf18@mails.tsinghua.edu.cn (Y.C.); liuxuan@tsinghua-hf.edu.cn (X.L.); xuh19@mails.tsinghua.edu.cn (H.X.)

**Keywords:** polypropylene, thermo-oxidative aging, reactive molecular dynamics, the reactive force field (ReaxFF), acetone, acetic acid

## Abstract

Understanding the aging mechanism of polypropylene (PP) is fundamental for the fabrication and application of PP-based materials. In this paper, we present our study in which we first used reactive molecular dynamics (RMD) simulations to explore the thermo-oxidative aging of PP in the presence of acetic acid or acetone. We studied the effects of temperature and oxygen on the aging process and discussed the formation pathways of typical small molecule products (H_2_, CO, CO_2_, CH_4_, C_2_H_4_, and C_2_H_6_). The effect of two infection agents, acetic acid and acetone, on the aging reaction was analyzed emphatically. The simulation results showed that acetone has a weak impact on accelerating the aging process, while acetic acid has a significant effect, consistent with previous experimental studies. By tracking the simulation trajectories, both acetic acid and acetone produced small active free radicals to further react with other fragment products, thus accelerating the aging process. The first reaction step of acetic acid is often the shedding of the H atom on the hydroxyl group, while the reaction of acetone is often the shedding of the H atom or the methyl. The latter requires higher energy at lower temperatures. This is why the acceleration effect of acetone for the thermo-oxidative aging of PP was not so significant compared to acetic acid in the experimental temperature (383.15 K).

## 1. Introduction

Polypropylene (PP) is one of the most promising thermoplastic polymers with excellent chemical stability, electrical insulation, and processing performance and is widely used in automobile, construction, and agriculture industries [1,2,3]. However, PP is prone to aging under heat, oxygen, light, and other conditions, resulting in its shape, color, and tensile strength undergoing irreversible changes, thus seriously affecting its long-term performance [4,5]. Among various external conditions, oxygen and temperature are the critical factors affecting aging. Therefore, it is necessary to study the thermo-oxidative aging mechanism for PP to predict its service life more reasonably.

Several experimental studies have focused on the aging process of PP. For example, Gugumus investigated the effect of temperature on the lifetime of PP films. They found that unstabilized PP films showed a marked downward curvature of the Arrhenius plot at temperatures below 80 °C, while phenolic antioxidant-stabilized PP films needed higher temperatures [6]. Hu et al. pointed out that the relative content of the carbon in a high oxidation state significantly increased when PP suffered from thermal-aging using X-ray photoelectron spectroscopy (XPS) [7]. Law et al. studied the effects of thermal aging on the microstructural changes of PP matrix in a composite using wide-angle X-ray scattering (WAXS) and Fourier-transform infrared spectroscopy (FTIR) [8]. They found the crystallinity of isotactic polypropylene increased with the aging process. Bernstein et al. carried out aging experiments on PP and detected the types of volatile products: aldehydes, ketones, acids, alcohols, esters, hydrocarbons, etc. [9,10].

Previous studies have proved that some small molecules with high reactivity produced in the aging process of polymers can accelerate the aging reaction as the infection agents. Sedlar et al. have long noted that the thermal-oxidative aging products of PP might in turn affect the stability of PP [11]. Eriksson et al. studied the spreading of oxidation in PP using imaging chemiluminescence (ICL), and found acetic acid increased the spreading rate of oxidation in PP [12]. Celina et al. studied the infection phenomenon under the condition of macroscopic distance and also found that acetic acid had a certain acceleration effect on the thermal-oxidative aging of PP [13,14,15]. In our previous experiments, we studied the accelerating effect of small molecules in the thermo-oxidative aging process of PP [16]. We found that different small molecules showed different infectious effects. Among them, acetic acid could significantly accelerate the aging process, while acetone had a weak accelerating aging effect. However, the thermo-oxidative aging process of PP involves many complex chemical reactions, and the aging mechanism cannot be elucidated at the atomic level only by experimental characterizations.

In traditional molecular dynamics (MD) simulation, the atoms are connected and fixed during the simulation process, both bond formation and bond dissociation cannot occur, and the chemical reactions cannot be described in the aging processes [17]. The quantum mechanics (QM) method has high calculation accuracy. It can provide a reaction mechanism in detail, but the computation burden hinders applying the QM method for complex polymer systems [18]. Compared with traditional MD and QM, the reactive molecular dynamics (RMD) simulation based on the reactive force field (ReaxFF) [19] might be the most suitable method for analyzing systems with complex chemical reactions. In the ReaxFF, the atoms are dynamically connected, determined by the real-time calculation of the atoms’ bond order. Therefore, the process of bond formation and dissociation can be well described. The ReaxFF has been successfully applied to various reaction systems, such as polymer [20,21,22,23,24,25,26], fossil fuels [27,28,29], energetic materials [30,31,32], combustion of small molecules [33,34], and transition metal catalysis [35]. Zhao et al. studied the pyrolysis of polycarbonate (PC). They found that C-O bond breakage of the terminal group or between PC monomers is the main reaction pathway of PC main chain breakage, the formation mechanism of significant products, such as CO_2_ and CO, which was also analyzed [36]. Yin et al. studied the effects of small molecule organic acids on the pyrolysis of meta-aramid (PMIA) fibers and found that formic acid could promote the decomposition of PMIA [37]. However, to our knowledge, few studies have focused on the accelerating effect of different small molecules on the aging process of polymer. There is no RMD simulation study of the PP thermo-oxidative aging mechanism based on the ReaxFF.

In this work, we applied RMD combing with the ReaxFF to study the thermo-oxidative aging mechanism of PP in the presence of acetic acid or acetone at the atomic level. We analyzed the crucial factors affecting the aging reaction, such as temperature and oxygen. Then, the typical aging products were calculated. The possible reaction pathways of acetic acid and acetone accelerating PP’s aging reaction were determined, and the differences in their effect on accelerating the aging process were also analyzed, combined with previous experimental results and free energy calculations. We hope this paper provides meaningful theoretical guidance for the mechanism of the thermo-oxidative aging of PP and the design of novel anti-aging PP materials.

## 2. Models and Methods

### 2.1. Models

**Figure 1:**
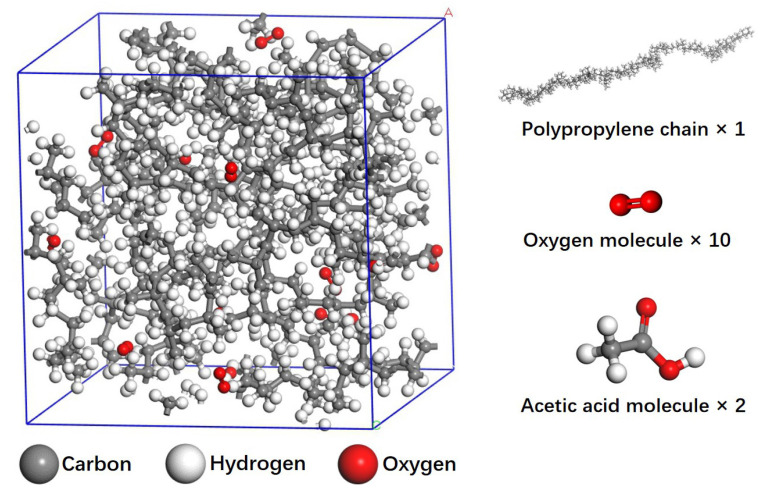
Configuration of PP-10O_2_-2C_2_H_4_O_2_ model (1 polypropylene (PP) chain, 10 oxygen molecules, and 2 acetic acid molecules were added into a box with periodic boundary conditions).

We first established the original PP models using Materials Studio 2018. Briefly, an isotactic amorphous PP chain (C_360_H_722_) with the degree of polymerization of 120 was placed in a box with periodic boundary conditions. A certain number of molecules of oxygen and acetic acid/acetone were randomly added into the box to obtain the aging model. In this work, the following models were built: PP-10O_2_, PP-10O_2_-2C_2_H_4_O_2_, PP-10O_2_-2C_3_H_6_O, PP-2C_2_H_4_O_2_, PP-100O_2_-2C_2_H_4_O_2_, PP-10O_2_-20C_2_H_4_O_2_, PP-10O_2_-20C_3_H_6_O, PP-10O_2_-10C_2_H_4_O_2_, PP-10O_2_-10C_3_H_6_O, PP-10O_2_-5C_2_H_4_O_2_, and PP-10O_2_-5C_3_H_6_O. The initial densities of these models were set as about 0.8 g · cm^–3^. The configuration of these models is shown in Figure 1.

### 2.2. The ReaxFF

ReaxFF is a bond order-based force field. The parameters in ReaxFF are derived from the comprehensive training set from QM calculations, maintaining a degree of precision. In ReaxFF, all energy terms representing atomic connectivity are associated with bond order, which is updated in real-time as the atoms’ position changes during the simulation. Therefore, the bond formation and dissociation can be characterized. The total potential energy of the system can be expressed as follows [19,38].

(1) $$E_{\mathrm{system}}=E_{\mathrm{bond}}+E_{\mathrm{lp}}+E_{\mathrm{over}}+E_{\mathrm{under}}+E_{\mathrm{val}}+E_{\mathrm{pen}}+E_{\mathrm{coa}}+E_{C2}+E_{\mathrm{trip}}\;+E_{\mathrm{tors}}+E_{\mathrm{conj}}+E_{H-\mathrm{bond}}+E_{\mathrm{vdWaals}}+E_{\mathrm{coulomb}}$$

where $E_{\mathrm{system}}$ represents the total system energy, including the bond energy $E_{\mathrm{bond}}$, long-pair energy $E_{\mathrm{lp}}$, over-coordination energy $E_{\mathrm{over}}$, under-coordination energy $E_{\mathrm{under}}$, valence angle $E_{\mathrm{val}}$, penalty energy $E_{\mathrm{pen}}$, three-body conjugation term $E_{\mathrm{coa}}$, correction for C2-molecule $E_{C2}$, triple-bond energy correction $E_{\mathrm{trip}}$, torsion angle term $E_{\mathrm{tors}}$, four-body conjugation term $E_{\mathrm{conj}}$, hydrogen bond interactions $E_{H-\mathrm{bond}}$, non-bonded van der Waals interaction $E_{\mathrm{vdWaals}}$, and electrostatic interaction $E_{\mathrm{coulomb}}$.

### 2.3. Simulation Details

Before the RMD simulation, geometry optimization was conducted to avoid unreasonable contacts among atoms in the original PP models. The Smart algorithm (a cascade of the steepest descent, adjusted basis set Newton-Raphson, and quasi-Newton methods) was adopted, in which the convergence limits of energy and force were 10^–4^ kcal mol^–1^ and 0.005 kcal mol^–1^·A^–1^, respectively. After geometry optimization, a 500 ps traditional MD calculation was conducted with the NPT ensemble (with constant particle number, pressure and temperature). The temperature and pressure were 323 K and 1.0 bar, respectively. The Berendsen thermostat and barostat were used with a decay constant of 0.1 ps. Then, a 200 ps traditional MD calculation was performed with the NVT ensemble (with constant particle number, volume and temperature) at 323 K using the Berendsen thermostat, and the decay constant was 0.1 ps. After the above steps, the density and energy of the system reached a stable value, indicating that the system had reached equilibrium. The final densities of all these models were consistent with the reported experiments (0.8~0.9 g cm^–3^).

The RMD simulations of initial structures obtained by the above steps were conducted using the LAMMPS platform (Stable version 7 August 2019). We used the NVT ensemble and the ReaxFF. The Nose–Hoover thermostat was used with a decay constant of 0.2 ps. The system was first balanced at 100 K for 12.5 ps and then heated from 100 K to a specified temperature (1600 K, 2000 K, 2400 K, 2500 K, and 3000 K) within 75 ps. Then, 500 ps RMD simulations were conducted at the specified temperature. After the above steps, the system was cooled to 1000 K within 25 ps and maintained at 1000 K for 25 ps. The time step of the RMD simulations was 0.25 fs. The intermediates and products during the simulation were recognized using a bond-order cutoff value of 0.2.

It should be noted that our previous accelerated aging research was conducted in the oven at 383.15 K [16]. However, the RMD simulation studies the conformational changes and reactions in molecules over very short time scales. If the simulation temperature is set to approximate the experimental temperature, it is difficult to observe the occurrence of reaction in the simulation of a short time. In the RMD simulation, we used high temperatures (e.g., 1600 K, 2000 K, 2400 K and 3000 K) to accelerate the aging process, so that the reaction could be seen in the short simulation time. Moreover, several studies have proved that despite the time and temperature differences, ReaxFF simulation and experiment results can still achieve excellent consistency [24,25,29,36,39,40].

### 2.4. Analysis Method of Reactants and Products

In the RMD simulation process, the initial PP chain cracks and reacts. More and more product fragments with low molecular weights occur, and both the kinds and number of product fragments in the system are continually increasing. Assuming that there are m kinds of fragments in the system at a specific moment, the ith product fragment is denoted as $F_{i}$, (I = 1, 2, …, m), and the number of the fragment $F_{i}$ is designated as $N_{i}$. The total number of fragments, $N_{t}$, is the sum of all fragments:

(2) $$N_{t}=\overset{m}{\underset{i=1}{\sum }}N_{i}$$

Oxygen is consumed continuously in the process of RMD simulation. Here, we investigated the average consumption rate of oxygen in the whole simulation stage. When analyzing the results, the change curve of the number of oxygen molecules with time was plotted, and the slope was obtained by linear fitting of the whole simulation time curve. If the oxygen was wholly consumed at one point before the end of the simulation, the curve from the beginning of the simulation to this point was selected for linear fitting. A higher absolute value of the slope means a faster average consumption rate of oxygen.

### 2.5. The Calculation of the Gibbs Free Energy Changes

To analyze the difficulties of the typical reactions detected in the simulation, Gaussian 09 was used to calculate the Gibbs free energy changes (ΔG) of the reactions. When calculating the Gibbs free energy, the total electron energy was obtained at M062X/6-311g(d, p), and the frequency analysis was performed at B3LYP/6-31g(d, p) to get the thermodynamic correction [41,42]. Compared with frequency analysis, the total electron energy calculation requires higher computational accuracy and has higher sensitivity to the basis set; in other words, it requires a larger basis set. For each reaction, ΔG at different temperatures were calculated: room temperature (298.15 K), experimental temperature (383.15 K), and simulation temperature (1600 K, 2000 K, 2400 K, 3000 K).

## 3. Results and Discussion

### 3.1. Effects of Temperature on the Aging of PP

As an essential factor of reaction thermodynamics, the temperature has an obvious significance on PP’s thermo-oxidative aging. For the three different systems (PP-10O_2_, PP-10O_2_-2C_2_H_4_O_2_, PP-10O_2_-2C_3_H_6_O), RMD simulations for 500 ps of thermo-oxidative aging at 1600 K, 2000 K, 2400 K, and 3000 K were conducted. The detailed simulation process is described in Section 2.3. The aging characteristics of these three systems have a similar variation tendency with the change of temperature. Here, we take the PP-10O_2_-2C_2_H_4_O_2_ system as an example to illustrate the effects of temperature on PP’s aging.

**Figure 2:**
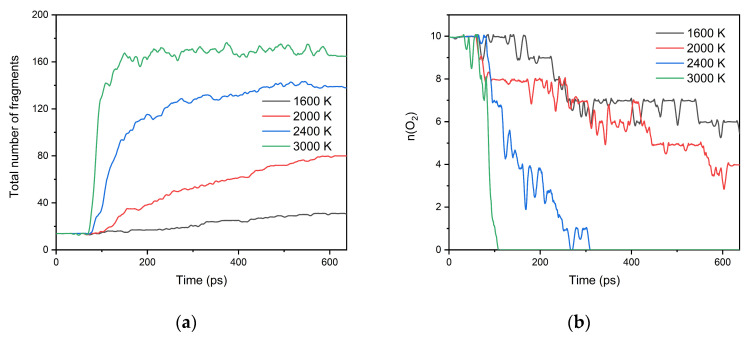
Changes of (**a**) the total number of fragments and (**b**) the number of oxygen molecules as a function of time at different simulation temperatures.

Figure 2 shows the variation of the total number of fragments and the number of oxygen molecules with time at different temperatures. As the simulation goes on, the total number of fragments gradually increases, indicating the PP chain breaks up and continuously decomposes into various small fragments. With the increase of temperature, the total number of fragments increases significantly, the average consumption rate of oxygen becomes faster. At 2400 K and 3000 K, the oxygen molecules can be consumed entirely at 305.8 ps and 105.0 ps, respectively. These indicate that the higher the temperature is, the faster the aging rate and the deeper the aging degree are.

**Figure 3:**
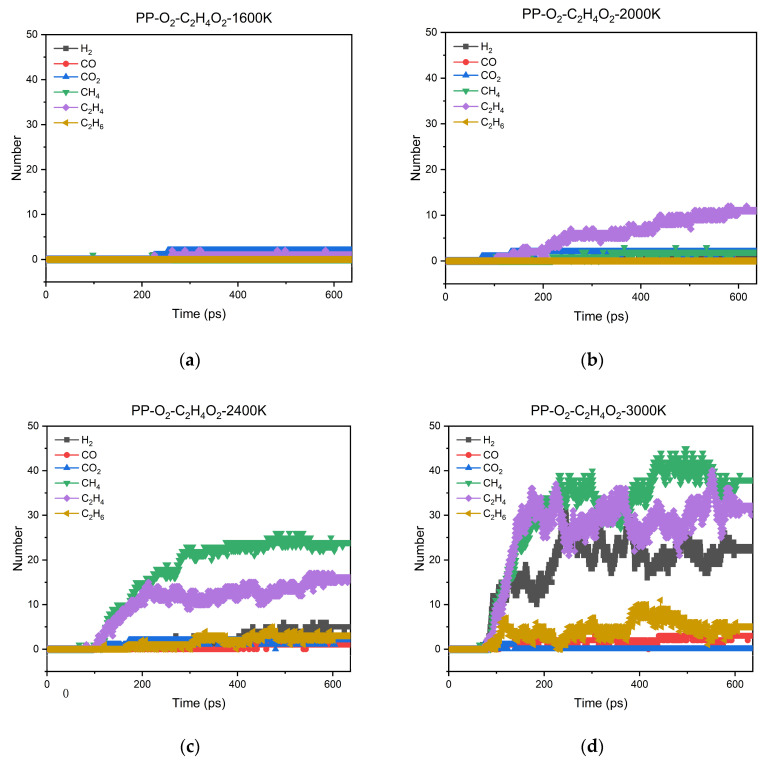
Evolution of six typical small molecule products as a function of time in the aging simulation: (**a**) 1600 K; (**b**) 2000 K; (**c**) 2400 K; (**d**) 3000 K.

In our previous experiments, we found that typical small molecules, such as H_2_, CO, CO_2_, CH_4_, C_2_H_4_, and C_2_H_6_, were detected in the aging reaction products of PP [16]. Figure 3 shows the changes in the numbers of these six typical small molecule products over time at different temperatures (1600 K, 2000 K, 2400 K, and 3000 K). It can be further seen that increasing the temperature deepens the degree of aging, so that the production of various small molecules gradually increases.

**Figure 4:**
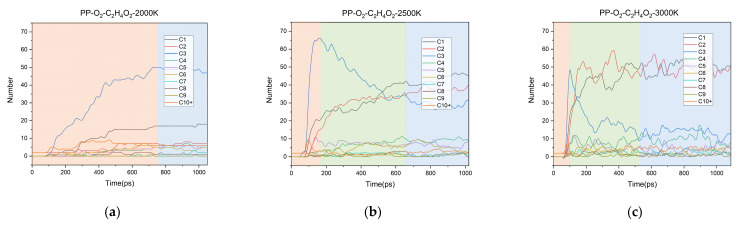
Distribution of major products at: (**a**) 2000 K; (**b**) 2500 K; (**c**) 3000 K.

With the increase of simulation time, the changes of products with different carbon numbers over time were counted at different simulation temperatures (2000 K, 2500 K, 3000 K). In Figure 4, C1 to C9 represent the products containing 1 to 9 carbon atoms, respectively, and C10+ represents the products containing 10 or more carbon atoms. It shows that the aging process can be divided into three stages: In the initial stage (orange region), C3 (mainly C_3_H_6_) first goes through a rapid growth process, which indicates rapid depolymerization of PP chain. In the intermediate stage (green region), with the deepening of aging, C3 is further consumed, the number of C1 and C2 products continue to increase and finally dominate. In the stable stage (blue region), the number of each component changes little and tends to balance. At higher temperatures, both the generation and consumption of C3 are more rapid, and the final production of C1 and C2 is also larger. The proportion of the final products is shown in Table S1.

### 3.2. Effects of Oxygen on the Aging of PP

Oxygen is also a major factor affecting the aging of polymer materials [4]. Here, we studied the PP aging under different oxygen content. We changed the molar ratios between C_2_H_4_O_2_ and O_2_ to 1:5 and 1:50, which were denoted as PP-10O_2_-2C_2_H_4_O_2_ and PP-100O_2_-2C_2_H_4_O_2_, respectively. The oxygen-free model PP-2C_2_H_4_O_2_ was also built. Other simulation conditions were unchanged, and the simulations were conducted at 3000 K.

**Figure 5:**
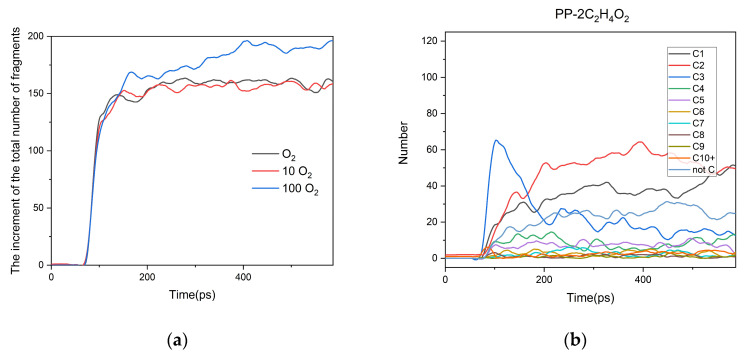

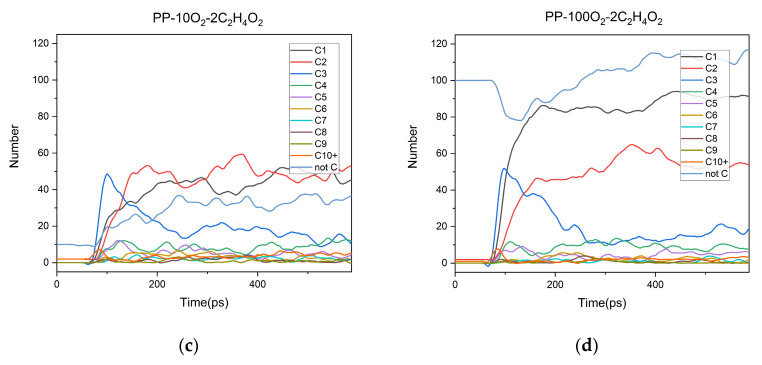
(**a**) The increment of the total number of fragments at different oxygen levels. The distribution of major products at (**b**) no O_2_; (**c**) 10 O_2_; (**d**) 100 O_2._

As shown in Figure 5a, the number of molecular fragments increased more significantly in the aging process of the PP-100O_2_-2C_2_H_4_O_2_ system, and they were not affected by the oxygen number, especially at the initial aging stage, indicating that the PP aging initiation is mainly related to temperature, rather than oxygen concentration. Figure 5b–d shows the distribution of major products in the three systems. It can be seen that in the aging process of the PP-2C_2_H_4_O_2_ system, the peak value of C3 molecules was higher than that of the other two systems. Further analysis of the output files shows that the rapid decomposition of long-chain hydrocarbons into C_3_H_6_ often occured in the PP-2C_2_H_4_O_2_ system. In the PP-100O_2_-2C_2_H_4_O_2_ system, all the non-carbon compounds (not C) were O_2_ at first. In the subsequent aging process, O_2_ was gradually consumed completely, while a large amount of H_2_O was generated. Furthermore, the number of C1 was also significantly increased, mainly because of the formation of a large amount of CO. The primary generation pathways of H_2_O and CO were analyzed, and the results are summarized in Table S2. It was found that H_2_O and CO are mainly obtained through the decomposition of fragments containing C, H, and O elements (C-H-O fragments).

Figure S1a summarizes the most common pathway, in which O_2_ participates in the aging reaction. Figure S1b,c shows the changes in the number of O_2_ products, the fragments containing C, H, and O elements, the fragments containing C and O elements, and the fragments containing H and O elements with time during the aging process of PP-10O_2_-2C_2_H_4_O_2_ and PP-100O_2_-2C_2_H_4_O_2_ systems. It can be seen that at the beginning of the reaction, O_2_ tended to combine with the fragments containing C and H elements produced by chain decomposition to form the fragments containing C, H, and O elements. In a system with high oxygen concentration, O_2_ is more inclined to create the fragments containing C and O elements and the fragments containing H and O elements as the reaction proceeds.

### 3.3. Formation Mechanism of Typical Small Molecule Products

Taking the PP-10O_2_-2C_2_H_4_O_2_ system as an example, we statistically analyzed the sources of six typical small molecule products (shown in Figure 3) based on the output file of the RMD simulation. The results are summarized in Table 1. CO was primarily generated by the breaking down of the carbon chains, and it can also be generated by the transformation of CO_2_. CO_2_ mainly comes from the decomposition of C_2_H_3_O_2_·, which is an intermediate product of acetic acid decomposition. C_2_H_6_ is mainly created in the following ways: the breaking down of the carbon chains, C_2_H_5_· capturing H, ethylene hydrogenation, and the combination of two CH_3_·. As shown in Figure 3, the amount of the above three small molecular products (CO_2_, CO, and C_2_H_6_) was relatively low compared with H_2_, CH_4_, and C_2_H_4_.

H_2_ can be formed through H_2_ breaking away from fragments containing C element, H· radical capturing H atom, and each of the two fragments providing one H atom. The sources of CH_4_ formation mainly include the breaking down of the carbon chains, CH_3_· capturing H atom, and H atom breaking away from CH_5_·. C_2_H_4_ is primarily generated by the breaking down of the carbon chains, C_2_H_3_· capturing H atom, H atom breaking away from C_2_H_5_· and ethanol dehydration.

**Figure 6:**
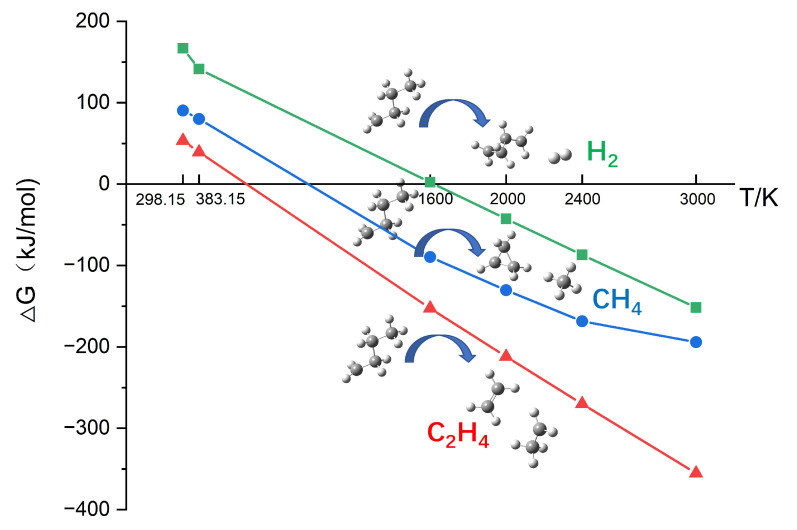
The Gibbs free energy changes at different temperatures: The generation of C_2_H_4_, CH_4_, and H_2._

As shown in Figure 3, it is interesting that, with the increase of temperature, the appearance of C_2_H_4_, CH_4_, and H_2_ can be detected successively. This phenomenon suggests that there may be differences in the difficulty of producing the three small molecules. Table 1 shows that the most common pathway of the formation of C_2_H_4_, CH_4_, and H_2_ was the breaking down of the fragments containing C element. Therefore, taking the reactions of C_4_H_9_· decomposing into C_2_H_4_, CH_4_, and H_2_ as examples, the ΔG values of these three reactions were calculated. Figure 6 shows the changes in ΔG with temperature for these three reactions. The values of ΔG are listed in Table S3. At the same temperature, it took the most energy for C_4_H_9_· to decompose into H_2_, followed by decomposing into CH_4_, and C_2_H_4_ took the least energy, which shows how hard the reaction is. On the other hand, the decomposition of C_4_H_9_· into C_2_H_4_ can proceed spontaneously at lower temperatures. Therefore, as the temperature rises, C_2_H_4_ is first observed, then is CH_4_, and finally, H_2_. The appearing sequence, as mentioned above, is also consistent with the phenomenon in Figure 3.

### 3.4. Reaction Mechanism for Acetic Acid and Acetone

Our previous research on PP film aging using Fourier-transform infrared spectroscopy (FTIR) showed that both acetic acid and acetone could accelerate the thermo-oxidative aging of PP, but acetone’s accelerating effect was weak, while acetic acid’s accelerating impact was more significant [16]. In this paper, the accelerating effect of acetic acid and acetone on the aging of PP was analyzed by the RMD simulation.

**Figure 7:**
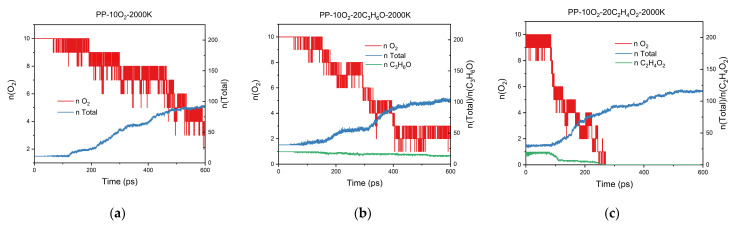
Evolution of the total number of molecular fragments and the number of oxygen molecules as a function of time: (**a**) PP-10O_2_—2000 K; (**b**) PP-10O_2_-20C_3_H_6_O—2000 K; (**c**) PP-10O_2_-20C_2_H_4_O_2_—2000 K.

The PP-10O_2_-20C_2_H_4_O_2_ model and the PP-10O_2_-20C_3_H_6_O model were established by increasing the number of acetic acid or acetone from 2 to 20. The RMD simulations of these two models and the PP-10O_2_ model were conducted at 2000 K. As shown in Figure 7, the simulation results of these three different systems were different. Acetone could accelerate the consumption of oxygen molecules, but not significantly, while acetic acid showed a significant acceleration effect and could make the oxygen wholly consumed. Furthermore, more molecular fragments were generated in the system with acetic acid added. These results showed that acetic acid had a more substantial acceleration effect on the thermo-oxidative aging of PP than acetone. The RMD simulation results have an excellent consistency with the experimental results.

**Figure 8:**
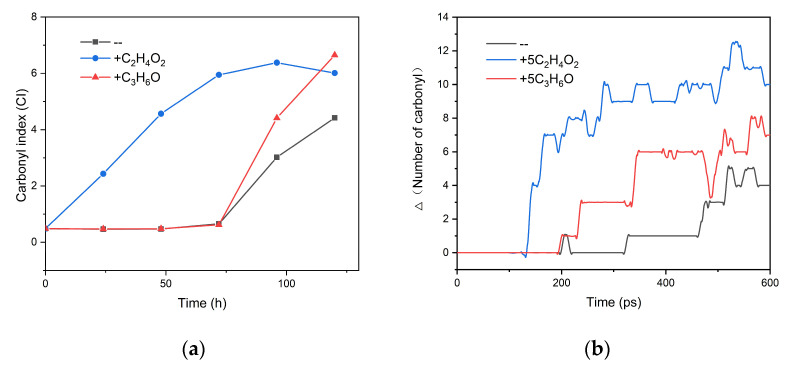
(**a**) The experimental results according to Reference [16]; (**b**) The reactive molecular dynamics (RMD) simulation result: evolution of the change of carbonyl number with time when the number of acetic acid/acetone added to the model is five (since acetic acid and acetone contained carbonyl groups, the number of carbonyls counted here should subtract the initial carbonyl number in the model for a more reasonable comparison).

Carbonyl index (CI) is an important parameter to characterize the aging degree of polymer, reflecting the number of carbonyl functional groups in samples. Figure 8a shows the experimental data from our previous research, which showed the evolution of CI with time under three different thermo-oxidative aging conditions: no small molecules added, acetone added, and acetic acid added. In this paper, keeping other conditions unchanged, the numbers of acetic acid or acetone added into the model were set as 2, 5, 10, and 20. After the RMD simulation at 2000 K, the variation of carbonyl number over time was calculated.

As shown in Figure S2, with the increase of acetic acid or acetone, the contribution of acetic acid in promoting the formation of the carbonyl was more and more obvious than that of acetone. When the number of acetic acid or acetone was 5, the obtained result was the closest to the experimental work, as shown in Figure 8. Our previous aging experiment was carried out at 110 °C, and 4.0 cm × 1.5 cm unstable PP sheet film and 20 μL liquid small molecules (acetone or acetic acid) were used [16]. Figure 8 shows that, for RMD simulation at 2000 K when the ratio of PP (degree of polymerization = 120) to small molecules is 1:5, similar aging conditions can be established as the experiment. According to Figure S2, it can also be predicted that in the aging experiment at 110 °C, if the amount of small molecules is increased to more than 20 μL, the difference in the acceleration effect between acetic acid and acetone will be more obvious.

**Figure 9:**
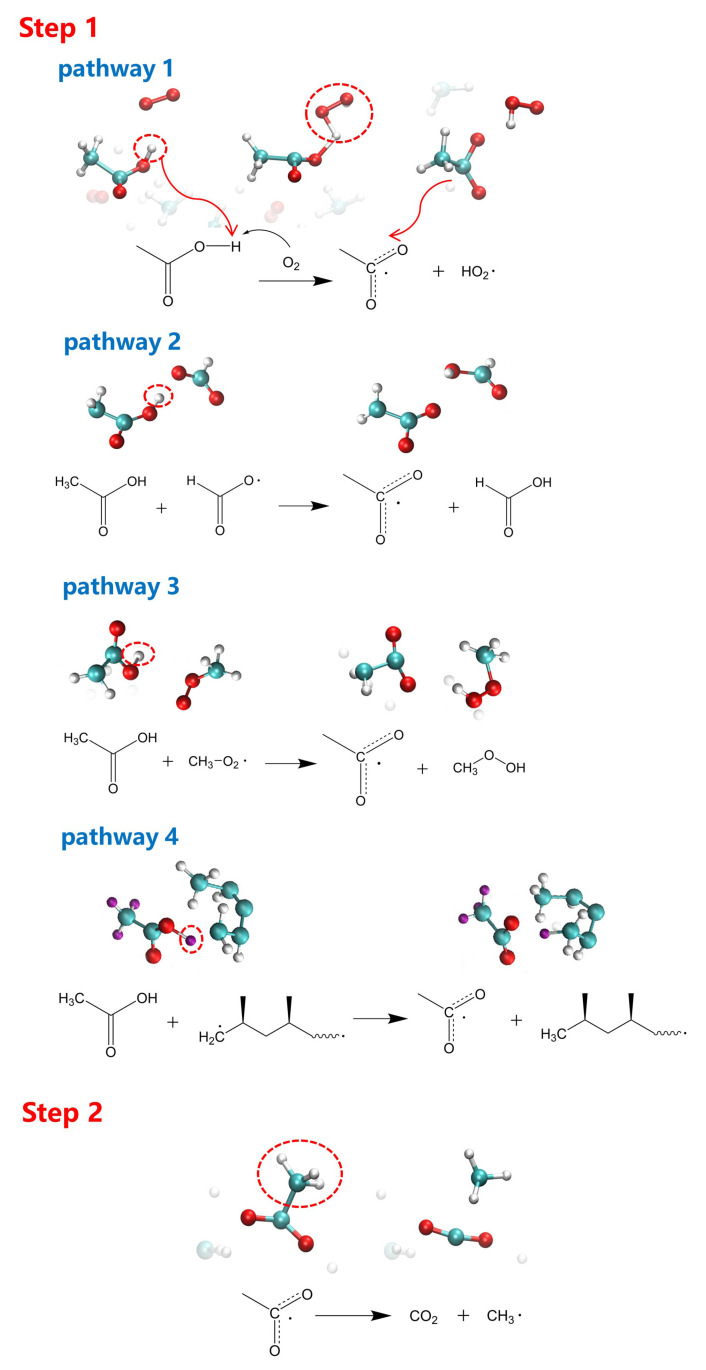
Main reaction paths of acetic acid during the aging process.

To further analyze the mechanism of the difference between acetic acid and acetone in accelerating the aging process, the specific pathways of their participation in the aging reaction were investigated. For the convenience of tracking the trajectory of small molecules in Visual Molecular Dynamics (VMD) [43], models of adding two molecules of acetic acid/acetone were adopted for analysis. The reaction pathways of acetic acid and acetone at different simulation temperatures (2000 K, 2500 K, and 3000 K) were tracked. The reaction pathways of acetic acid in the models with varying concentrations of oxygen mentioned in Section 3.2 were also analyzed. Typical reaction pathways of acetic acid are shown in Figure 9.

Step 1: The H atom on the hydroxyl group in acetic acid was taken away, resulting in the formation of intermediate C_2_H_3_O_2_· radical. At a higher temperature (3000 K) or higher oxygen concentration, the H atoms tended to be captured by O_2_ (pathway 1). When the temperature was relatively low (2000 K and 2500 K), the H atoms tended to be captured by fragments containing the carbon element. These fragments included: another acid that is lacking the H atom on the hydroxyl group (pathway 2), the fragment combining O_2_ and small free radicals such as CH_3_· (pathway 3), and long carbon chains (pathway 4).

Step 2: the C_2_H_3_O_2_· intermediate radical continued to decompose into CO_2_ and CH_3_·.

In short, the most typical pathway for acetic acid to participate in the aging reaction can be summarized as follows. First, the H atom on the hydroxyl group is taken away, and then the remaining C_2_H_3_O_2_· decomposes to produce CO_2_ and CH_3_·. In addition to the most common reactions mentioned above, other pathways were also observed and are described below. For example, the H atom on the methyl group of acetic acid can be taken away first, and then the remaining C_2_H_3_O_2_· continues to decompose into OH· radical and C_2_H_2_O fragment. Acetic acid can also capture the H and O atoms from two HO_2_· radicals to form C_2_H_5_O_3_·. The snapshots of these reactions are summarized in Figure S3.

Figure S4 shows the change of relative energy at 3000 K for the typical reaction paths of acetic acid. For the first step reaction of acetic acid, more energy is necessary for O_2_ capturing the H atom than other fragments capturing the H atom, which explains why the reaction of O_2_ capturing the H atom was not detectable at lower simulation temperatures. After acetic acid loses the H atom on the hydroxyl group, the remaining C_2_H_3_O_2_· decomposes into CH_3_· and CO_2_. As a result of this reaction path, we observed that the energy significantly reduced (–391.73 kJ/mol), indicating that this reaction was elementary to occur.

**Figure 10:**
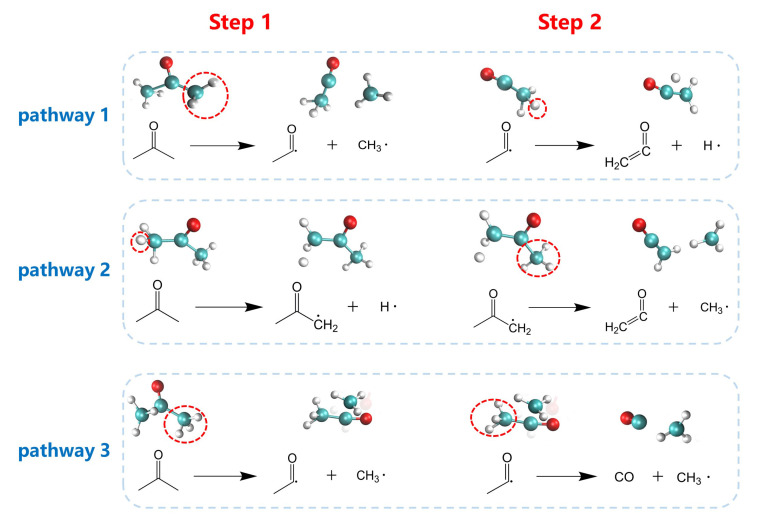
Major reaction pathways of acetone during the aging process.

Typical reaction paths of acetone are shown in Figure 10 and their general behavior is described as follows. The H atom breaks away from the acetone first, then the CH_3_· radical breaks away, and forms a C_2_H_2_O fragment (pathway 1). The order of the two steps can be swapped. The CH_3_· radical breaks away from the acetone first, then the H atom breaks away, and forms the C_2_H_2_O fragment (pathway 2). Furthermore, two CH_3_· can also break away from the acetone successively to form CO (pathway 3). It should be noted that acetone does not participate in the aging reaction at 2000 K, which further indicates that acetone is difficult to participate in the aging reaction when the temperature is low.

Figure S5 shows the change of relative energy at 3000 K for the typical reaction paths of acetone. It was found that the breaking away of CH_3_· from acetone occurred more easily than the breaking away of H·. The pathway 3, where two CH_3_·s broke away from the acetone successively to form CO, showed the largest energy decline, which was 170.36 kJ/mol more than pathway 1 and pathway 2.

**Figure 11:**
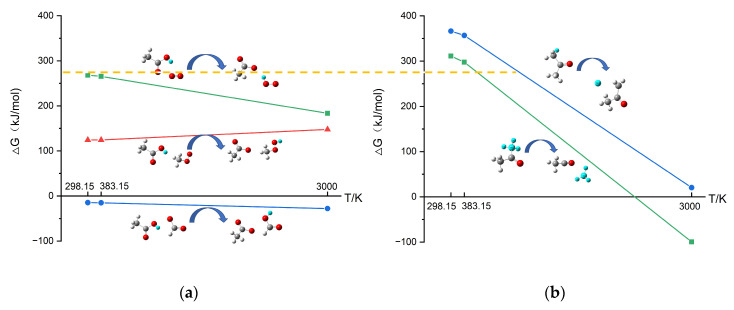
Gibbs free energy change for the first step reactions of acetic acid and acetone at different temperatures: (**a**) acetic acid; (**b**) acetone.

The above research results showed that when acetone and acetic acid participate in PP’s thermo-oxidative aging reaction, some small free radicals with high activity (CH_3_, H, etc.) are generated, which further react with other fragments, thus accelerating the aging process. The results also showed that acetic acid’s first step reaction was usually the shedding of the H atom on the hydroxyl group. In contrast, acetone’s first step reaction was usually the shedding of H or CH_3_. The ΔG of these typical first step reactions at different temperatures were calculated using Gaussian 09, as shown in Figure 11. All the values for specific reactions of acetic acid and acetone at different temperatures mentioned above can be found in Tables S4 and S5.

When the temperature was low, close to the room temperature (298.15 K) and experimental temperature (383.15 K), the first step reaction for acetone generally required higher energy than acetic acid. Therefore, in the experimental temperature condition, the acetone is harder to involve in the aging reactions, and its acceleration effect is not obvious compared to acetic acid. This result is consistent with the experimental result.

## 4. Conclusions

In this paper, the ReaxFF MD simulation combined with the quantum mechanics (QM) method were first used to investigate the thermo-oxidative aging mechanism for polypropylene under the influence of acetic acid and acetone in the aging process.

The whole aging process can be divided into three stages: in the initial stage, C3 species is massively generated by the rapid depolymerization of the PP chain; in the intermediate stage, C3 is consumed, while the number of C1 and C2 increases; in the stable stage, the number of each component tends to balance. As the simulation temperature increases, the aging process is accelerated, more fragments occur, and the oxygen consumption rate becomes faster. With the increase of oxygen concentration, the aging decomposition of PP is promoted, but the rapid aging reaction’s initiation time is not affected. Additionally, the system tends to produce large amounts of H_2_O and CO at high oxygen concentration. The main formation pathways were summarized for the typical small molecule products (H_2_, CO, CO_2_, CH_4_, C_2_H_4_, C_2_H_6_) detected in the experiment. Among them, H_2_, CH_4_, and C_2_H_4_ had higher production. With the increase of temperature, the appearance of C_2_H_4_, CH_4_, and H_2_ could be detected successively. By calculating ΔG, it was found that the reaction to produce C_2_H_4_ could proceed spontaneously at a lower temperature, while the reaction to produce CH_4_, especially H_2_, needed higher temperatures.

Then the reaction mechanism for acetic acid and acetone was studied. The phenomenon observed in the experiment was reproduced by simulation: Acetone had a weak effect on accelerating the aging process, while acetic acid had a significant impact. Both acetic acid and acetone generate small free radicals with high activity (CH_3_, H, etc.) to further react with other fragments, thus accelerating the aging process. For acetic acid, first, the H atom on the hydroxyl group was easily captured by O_2_ or other fragments containing C element, and then, the remaining C_2_H_3_O_2_· decomposed to CO_2_ and CH_3_·. For acetone, CH_3_· and the H atom from another CH_3_· broke away successively to form C_2_H_2_O, or two CH_3_·s broke away successively to form CO. Further calculations revealed that acetone’s first step reaction generally required higher energy than that of acetic acid when the temperature was low, which explains why the acceleration effect of acetone was not obvious compared to acetic acid in the experimental temperature (383.15 K). Based on previous studies on the infection phenomenon of PP aging [11,12,13,14,15,16] and this work, for different small molecular infection agents, we believe their acceleration effect on PP aging may depend on the following factors: the difficulty of their initial reaction and their ability to generate active free radicals. In practical applications, it is necessary to pay more attention to the infection agents with a strong acceleration effect and make reasonable prediction of their infection behavior, so as to guide the design and reasonable use of PP materials, and prolong the life of PP materials.

These results verify that the RMD simulation based on ReaxFF is an effective method to analyze the microscopic mechanism of thermo-oxidative aging of PP. It has a broad application prospect in predicting the service life of polymer materials.

## Figures and Tables

**Table 1:** The formation methods of six typical small molecule.

Product	Generation Ways	The Proportion of Generation Way	Reactions
CO	Carbon chains break down	83.33%	$C_{6}H_{5}O\cdot \to \mathrm{CO}+C_{5}H_{5}\cdot$
	O atom breaks away from CO_2_	16.67%	${\mathrm{CO}}_{2}+H_{2}+C_{4}H_{10}\to \mathrm{CO}+C_{4}H_{12}O$
CO_2_	CO_2_ breaks away from C_2_H_3_O_2_·	66.67%	$C_{2}H_{3}O_{2}\cdot \to {\mathrm{CO}}_{2}+{\mathrm{CH}}_{3}\cdot$
	H atom breaks away from CHO_2_·	33.33%	${\mathrm{CHO}}_{2}\cdot +C_{17}H_{28}\to {\mathrm{CO}}_{2}+C_{17}H_{29}\cdot$
C_2_H_6_	Carbon chains break down	53.57%	$C_{3}H_{9}\cdot \to C_{2}H_{6}+{\mathrm{CH}}_{3}\cdot$
	C_2_H_5_· captures H atom	21.43%	$C_{2}H_{5}\cdot +C_{6}H_{8}\to C_{2}H_{6}+C_{6}H_{7}\cdot$
	ethylene hydrogenation	10.71%	$C_{2}H_{4}+H_{2}\to C_{2}H_{6}$
	two CH_3_· combinate	7.14%	$2{\mathrm{CH}}_{3}\cdot \to C_{2}H_{6}$
	others	7.14%	$C_{2}H_{6}O_{2}\to C_{2}H_{6}+O_{2}$
C_2_H_4_	Carbon chains break down	63.79%	$C_{3}H_{7}\cdot \to C_{2}H_{4}+{\mathrm{CH}}_{3}\cdot$
	C_2_H_3_· captures H atom	10.34%	$C_{2}H_{3}\cdot +H\cdot \to C_{2}H_{4}$
	H atom breaks away from C_2_H_5_·	12.07%	$C_{2}H_{5}\cdot \to C_{2}H_{4}+H\cdot$
	ethanol dehydration	8.62%	$C_{2}H_{6}O\to C_{2}H_{4}+H_{2}O$
	others	5.17%	${\mathrm{CH}}_{3}\cdot +C_{2}H_{6}O\to C_{2}H_{4}+{\mathrm{CH}}_{5}O\cdot$
CH_4_	Carbon chains break down	52.08%	$C_{4}H_{9}\cdot \to {\mathrm{CH}}_{4}+C_{3}H_{5}\cdot$
	CH_3_· captures H atom	35.42%	${\mathrm{CH}}_{3}\cdot +C_{3}H_{6}\to {\mathrm{CH}}_{4}+C_{3}H_{5}\cdot$
	H atom breaks away from CH_5_·	12.50%	${\mathrm{CH}}_{5}\cdot +C_{3}H_{6}\to {\mathrm{CH}}_{4}+C_{3}H_{7}\cdot$
H_2_	H_2_ breaks away from C fragment ^1^	46.88%	$C_{4}H_{9}\cdot \to H_{2}+C_{4}H_{7}\cdot$
	H· captures H atom	28.13%	${\mathrm{CH}}_{4}+H\cdot \to H_{2}+{\mathrm{CH}}_{3}\cdot$
	each of the two fragments provides one H atom	25.00%	$C_{6}H_{9}\cdot +C_{8}H_{10}\to H_{2}+C_{6}H_{8}+C_{8}H_{9}\cdot$

^1^ C fragments: Fragments containing the element carbon.

## Data Availability

The data presented in this study are available on request from the corresponding author.

## Supplementary Materials

The following are available online at https://www.mdpi.com/article/10.3390/polym13081243/s1, Figure S1: (a) Consumption ways of O_2_ at PP aging system. Distribution of oxygen-containing fragments at: (b) PP-10O_2_-2C_2_H_4_O2—3000 K (c) PP-100O_2_-2C_2_H_4_O_2_—3000 K, Figure S2: Evolution of the change of carbonyl number with time when the number of acetic acid/acetone added to the model was (a) 2; (b) 5; (c) 10; (d) 20, Figure S3: Other reaction paths for acetic acid, Figure S4: Gibbs free energy change (ΔG) for typical acetic acid reactions, Figure S5: Gibbs free energy change for typical acetone reactions, Table S1: Proportion of the final products, Table S2: The formation method of CO and H_2_O in the system with 100 O_2_, Table S3: Calculation results of ΔG at different temperatures for the generation reactions of H_2_, CH_4_, and C_2_H_4_, Table S4: Calculation results of ΔG at different temperatures for the typical reactions of acetic acid, Table S5: Calculation results of ΔG at different temperatures for the typical reactions of acetone.
