# Supplementary material for: How Small Molecules Affect the Thermo-Oxidative Aging Mechanism of Polypropylene: A Reactive Molecular Dynamics Study
Source: Polymers (Basel). 2021 Apr 12;13(8):1243. doi: 10.3390/polym13081243 (PMC8069144; doi:10.3390/polym13081243)
Supplement: Supplementary file 1 [file polymers-13-01243-s001.pdf]

Supplementary Information

How small molecules affect the thermo-oxidative aging mechanism of polypropylene: A reactive molecular dynamics study

Fan Zhang [1], Yufei Cao [1] , Xuan Liu [1] , Huan Xu [1] , Diannan Lu [1,*] and Rui Yang [1,*]

[1] Department of Chemical Engineering, Tsinghua University, Beijing 100084, China

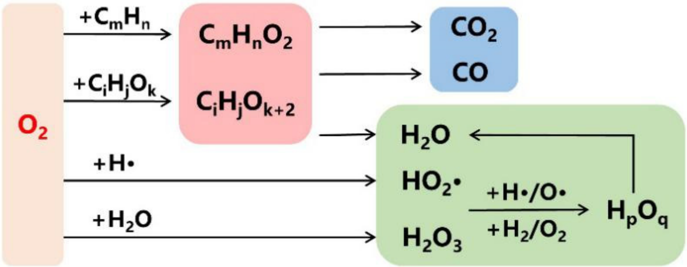

(a)

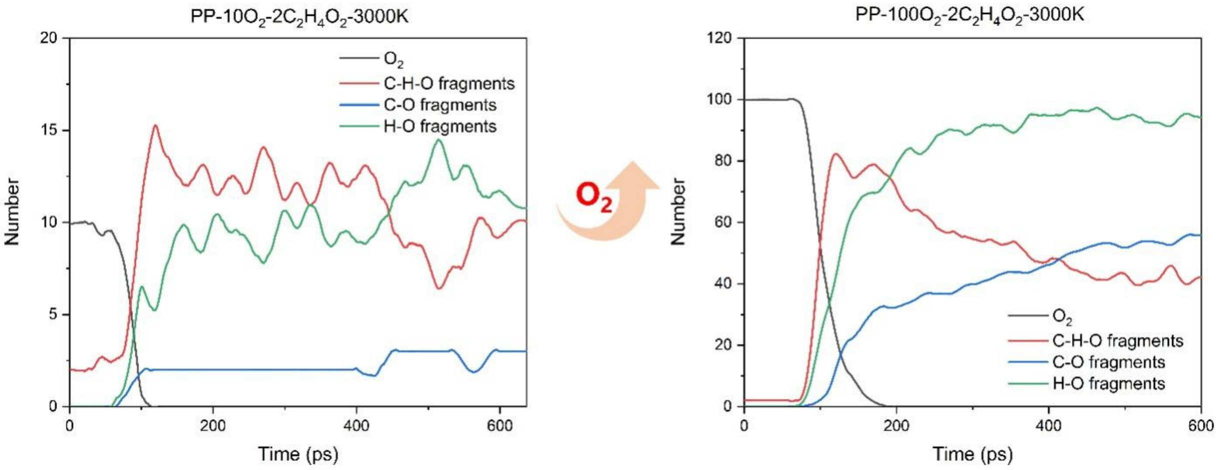

(b) (c)

Figure S1. (a) Consumption ways of O_2 at PP aging system; Distribution of oxygen-containing fragments at: (b)PP-10O_2-2$C_2H_4O_2$-3000 K (c)PP-100O_2-2$C_2H_4O_2$-3000 K

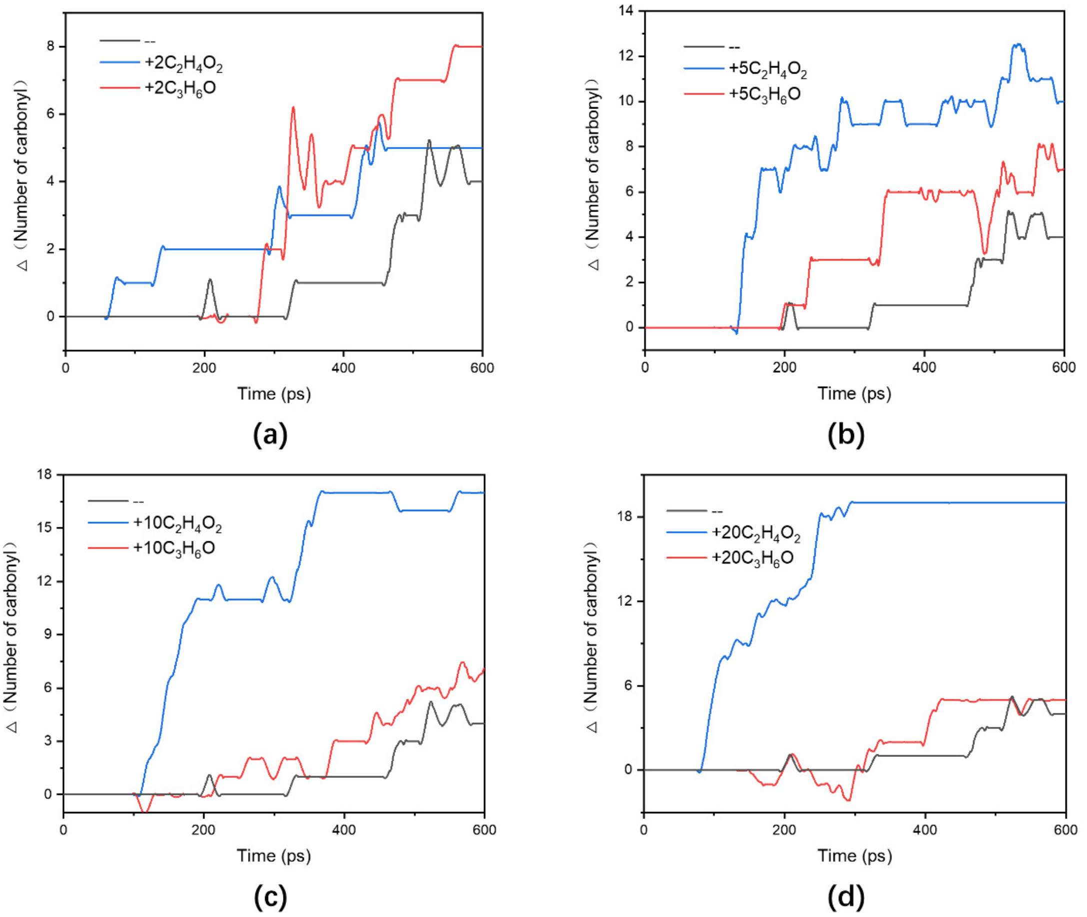

Figure S2. Evolution of the change of carbonyl number with time when the number of acetic acid / acetone added to the model was (a)2; (b)5; (c)10; (d)20.

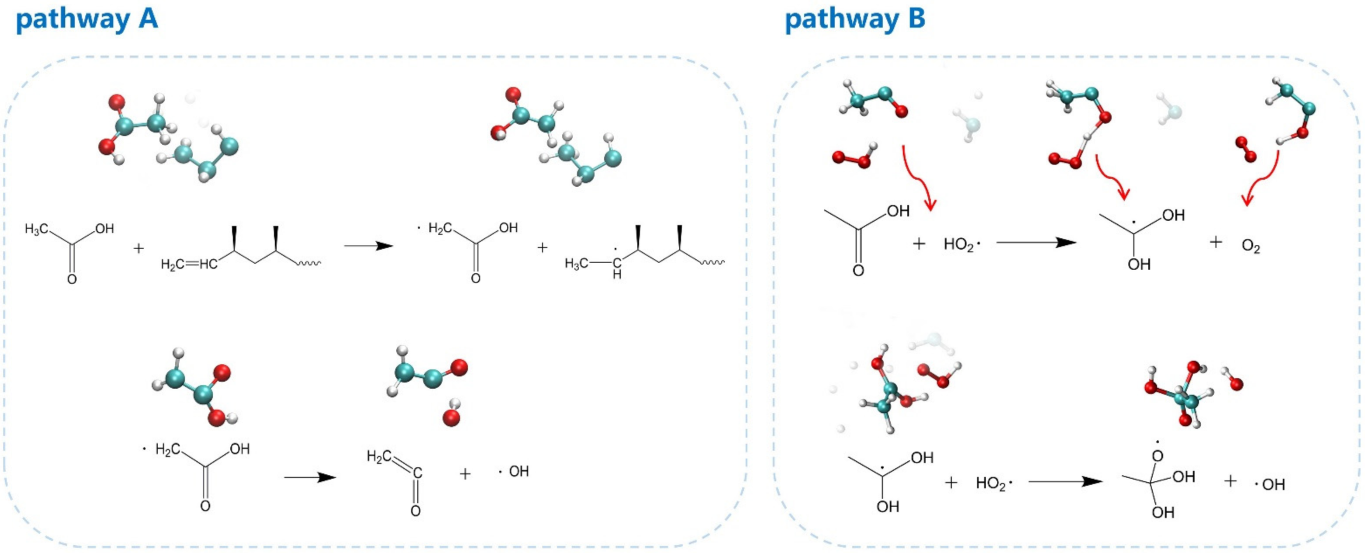

Figure S3. Other reaction paths for acetic acid

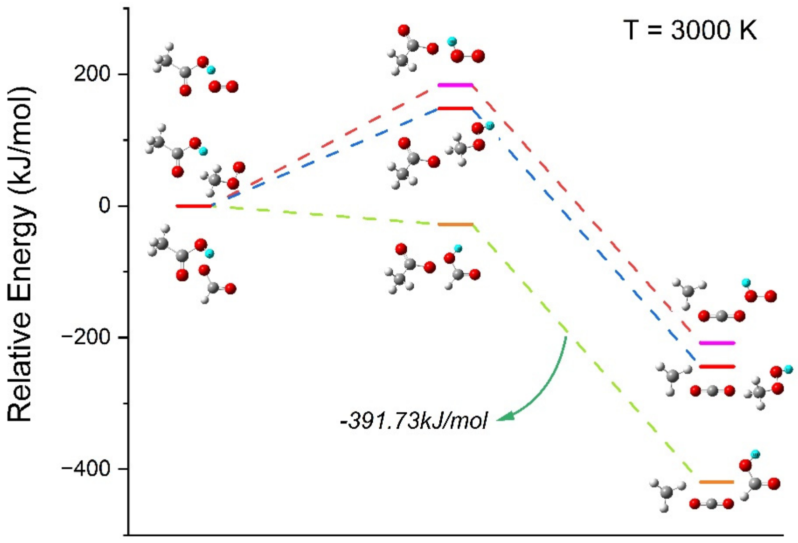

Figure S4. Gibbs free energy change for typical acetic acid reactions

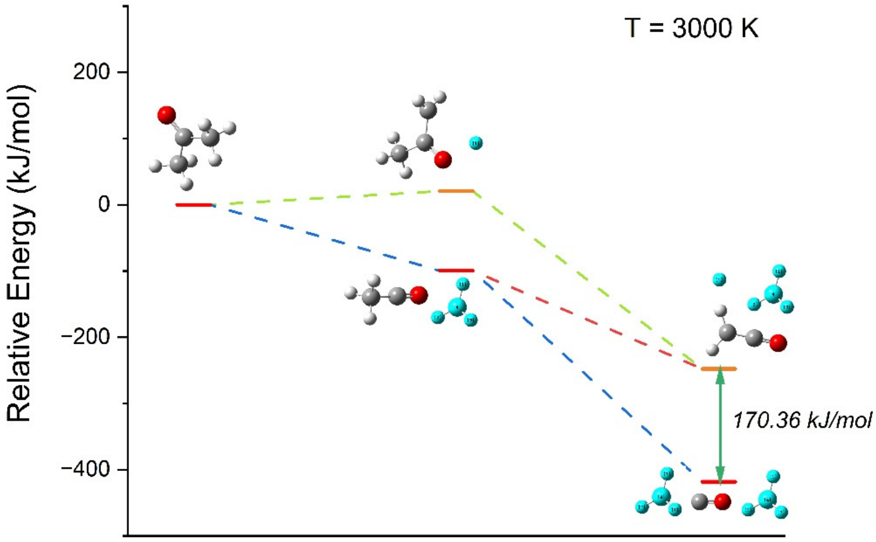

Figure S5. Gibbs free energy change for typical acetone reactions

Table S1. Proportion of the final products

T/K	C1	C2	C3	C4	C5	C6	C7	C8	C9	C10+	Not C	Total
2000	17.9%	6.9%	46.1%	5.9%	4.9%	4.9%	2.0%	1.0%	0	5.9%	4.9%	102
2500	28.8%	26.1%	17.0%	5.9%	3.3%	3.3%	1.3%	0	0	2.6%	11.8%	153
3000	30.2%	29.0%	8.3%	5.3%	2.4%	0.6%	0.6%	0.6%	0.6%	3.0%	19.5%	169

Table S2. The formation method of CO and H_2O in the system with 100 O_2

Product	Generation ways	The proportion of generation way	Reactions
CO	CO breaks away from C-H-O fragments [1](C≥2)	60.5%	$C_4H_3O\cdot \rightarrow CO + C_3H_3\cdot$
	H atom breaks away from CHO·	26.3%	$CHO\cdot \rightarrow CO + H\cdot$
	C-H-O fragments(C=1) break down into CO and H_mO	13.2%	$CHO\cdot + H_2O \rightarrow CO + H_3O\cdot$
H_2O	H_2O breaks away from C-H-O fragments	74.4%	$C_3H_7O\cdot \rightarrow H_2O + C_3H_5\cdot$
	H atom breaks away from H_2O_2	9.3%	$H_2O_2 + C_2H_2 \rightarrow H_2O + C_2H_2O$
	Other H_mO_n fragments decompose	16.3%	$H_2O_3 \rightarrow H_2O + O_2$

[1] C-H-O fragments: Fragments containing the elements carbon, hydrogen, and oxygen

Table S3. Calculation results of ∆G at different temperatures for the generation reactions of H_2, CH_4 and C_2H_4

Reaction	∆G (kJ/mol)					
	298.15 K	383.15 K	1600 K	2000 K	2400 K	3000 K
$C_4H_9\cdot \rightarrow H_2 + C_4H_7\cdot$	166.76	141.30	2.15	-42.82	-86.99	-151.86
$C_4H_9\cdot \rightarrow CH_4 + C_3H_5\cdot$	90.36	80.03	-89.60	-130.38	-168.66	-194.15
$C_4H_9\cdot \rightarrow C_2H_4 + C_2H_5\cdot$	53.31	39.29	-152.59	-212.01	-270.13	-355.31

Table S4. Calculation results of ∆G at different temperatures for the typical reactions of acetic acid

Reaction	∆G (kJ/mol)		
	298.15 K	383.15 K	3000 K
$CH_3COOH + O_2 \rightarrow CH_3COO\cdot + HO_2\cdot$	268.18	265.44	183.48
$CH_3COOH + CHO_2\cdot \rightarrow CH_3COO\cdot + CH_2O_2$	-14.66	-15.12	-27.90
$CH_3COOH + CH_3O_2\cdot \rightarrow CH_3COO\cdot + CH_4O_2$	124.41	124.44	147.77
$CH_3COO\cdot \rightarrow CO_2 + CH_3\cdot$	-101.61	-111.26	-391.73

Table S5. Calculation results of ∆G at different temperatures for the typical reactions of acetone

Reaction	∆G (kJ/mol)		
	298.15 K	383.15 K	3000 K
$CH_3COCH_3 \rightarrow CH_3CO\cdot + CH_3\cdot$	311.21	297.77	-99.44
$CH_3CO\cdot \rightarrow CH_2CO + H\cdot$	143.08	134.99	148.47
$CH_3CO\cdot \rightarrow CO + CH_3\cdot$	10.11	-0.53	-318.83
$CH_3COCH_3 \rightarrow CH_3COCH_2\cdot + H\cdot$	366.30	356.69	20.55
$CH_3COCH_2\cdot \rightarrow CH_2CO + CH_3\cdot$	87.99	76.06	268.45
